# Before it is too late: pre-emptive ablation strategies in tetralogy of Fallot

**DOI:** 10.1093/europace/euae064

**Published:** 2024-03-26

**Authors:** Mary C Niu, Thomas A Pilcher, Susan P Etheridge

**Affiliations:** Department of Pediatrics, Division of Cardiology, University of Utah and Primary Children’s Hospital, 81 Mario Capecchi Drive, Salt Lake City, UT 84112, USA; Department of Pediatrics, Division of Cardiology, University of Utah and Primary Children’s Hospital, 81 Mario Capecchi Drive, Salt Lake City, UT 84112, USA; Pediatric Cardiology, St. Luke’s Children’s Hospital, 305 E. Jefferson Street, Boise, ID 83712, USA

**This editorial refers to ‘Automated isochronal late activation mapping for substrate characterization in patients with repaired tetralogy of Fallot’, by E. Arana-Rueda *et al.*, https://doi.org/10.1093/europace/euae062**.

As the most common cyanotic congenital heart disease (CHD), tetralogy of Fallot (TOF) and its trajectory in the CHD historical landscape exemplify the intricate interplay between the natural and altered progression of CHD. Its narrative illuminates how our understanding of the disease, the interventions that modify it, and the decision-making for future directions are interrelated. This motif extends into the work presented by Arana-Rueda *et al.*^1^ in this issue of *Europace*.

In their work, Arana-Rueda *et al.*^1^ introduce an innovative and novel application of automated isochronal late activation mapping (ILAM) for substrate characterization and ablation in a small series of 14 patients with repaired TOF. While ILAM is an established technique for mapping and ablating adult ventricular tachycardia (VT),^2^ its use in CHD marks a significant departure from previous applications. In this study, the authors demonstrate that ILAM, obtained by high-density mapping, accurately identifies slow conducting anatomic isthmuses (SC-AIs)—defined as conduction velocity <0.5 m/s—in patients with repaired TOF. Specifically, deceleration zones—defined as regions with more than three isochrones within a 1 cm radius—were shown to correctly identify SC-AI < 0.5 m/s, with an area under the receiver operating characteristic curve of 99% (*P* < 0.001), 90% sensitivity, 100% specificity, and an accuracy of 0.94. Moreover, the presence of deceleration zones was significantly associated with VT inducibility (*P* = 0.006). These deceleration zones co-localized with the critical isthmus participating in 100% of inducible re-entrant VTs (with a total of eight macro–re-entry VT substrates). Two additional noteworthy findings warrant mention: first, there were no complications with this approach; second, the use of high-density mapping enabled the acquisition of a median of 2149 points over a relatively short procedure duration (median 140 min; interquartile range 133, 180). The reported findings strongly indicate that automated ILAM with high-density mapping effectively identifies deceleration zones crucial for re-entrant VT. As a result, the integration of this automated ILAM approach should be considered in the mapping and ablation of VT substrates in patients with repaired TOF.

Beyond the technological advancements presented, this work brings forth broader considerations from the historical landscape of TOF that frames ongoing discourse in the field. These considerations underscore the dynamic interplay between medical advancements and the evolving needs of patients with repaired TOF. Early surgical breakthroughs, while significantly improving survival,^3^ introduced long-term complications for the expanding TOF population, including pulmonary valve regurgitation, maladaptive right ventricular remodelling, and VT. Whether acting independently or synergistically, these factors collectively contribute to an elevated risk of sudden death within this patient cohort.

Subsequent studies sought to identify high-risk patients for sudden cardiac death and guide management with optimal timing of interventions including pulmonary valve replacement (PVR), implantable cardioverter-defibrillator implantation, and therapeutic catheter ablation targeting SC-AI VT substrates.^4,5^ Despite rationale suggesting that arrhythmia risk could be mitigated by addressing pulmonary regurgitation and eliminating haemodynamic burdens, PVR alone proves insufficient in preventing VT and sudden death.^6^

This is not surprising, as PVR does not eliminate SC-AIs between electrically inert anatomical and surgically induced barriers (e.g. valve annuli, prosthetic material, surgical, or acquired myocardial scars) that form the substrate for re-entry VT.^7–9^ Notably, while most TOF patients have anatomical isthmuses, only those with slow conduction (SC-AI) possess the arrhythmic substrate for VT. Identifying and interrupting these slowly conducting isthmuses have become crucial treatment targets to prevent VT, irrespective of inducibility. Ablation of these targets, demonstrating bidirectional block, can eliminate VT substrates even before VT occurs. Studies, exemplified by Kapel *et al.*^9^ in the *European Heart Journal*, underscore the success of ablation in preventing VT recurrence and ensuring a durable outcome.

Importantly, PVR, especially the newer versions of the transcatheter PVR in the native right ventricular outflow tract, may cover vital isthmuses, rendering them inaccessible and potentially making a future catheter ablation obsolete for patients who develop VT after PVR implantation. As such, a growing movement supports pre-procedural electrophysiology (EP) studies to eliminate latent ventricular arrhythmia substrates. Despite this, there remains significant uncertainty about the efficacy of pre-procedural ablation strategies in preventing future arrhythmias. Although the outcome of preventing future VT may be unknown, the data presented here are encouraging, and Arana-Rueda *et al.* provide an approach that does not add significant time or complications to traditional EP studies.

Against this backdrop, there are several additional noteworthy aspects from Arana-Rueda *et al.*’s study that significantly contribute to the broader discourse surrounding repaired TOF. First, the incorporation of routine VT substrate characterization as part of a pre-PVR procedure evaluation protocol reveals a high prevalence of SC-AIs and inducible VT in patients with repaired TOF. Ten of the 14 patients (71%) in the case series underwent VT substrate characterization as part of a routine evaluation protocol to systematically assess the risk for ventricular arrhythmias prior to PVR. Among these patients, 60% were identified with SC-AIs, and 50% exhibited inducible VT. This reported prevalence of 50% for inducible VT in pre-PVR patients is on the higher end but remains consistent with previous findings reporting 22–50% prevalence ranges for inducible VT in pre-PVR patients.^10,11^ These data highlight the importance of comprehensive risk assessment in the repaired TOF population.

Second, the localization of SC-AIs, particularly within isthmus 3 (i.e. the isthmus between the ventricular septal defect patch and the pulmonary valve), underscores the potential challenges in accessibility following PVR, as isthmus 3 is particularly susceptible to being rendered inaccessible after PVR. Among the 11 SC-AIs identified in this study, 10 localized to isthmus 3. This finding is consistent with prior studies that have identified isthmus 3 as the most commonly implicated isthmus for re-entry VT in repaired TOF.^10,11^ This observation adds a layer of complexity to the management of repaired TOF patients and raises questions about how to address the impact of PVR on these critical VT substrates.

Third, successful ablation targeting isthmus 3 and achieving conduction block in the associated deceleration zones demonstrate promising outcomes in preventing VT. When isthmus 3 was targeted in ablations, conduction block through deceleration zones in isthmus 3 and non-inducibility was successfully achieved in all cases (*n* = 6); VT was not observed in any of these cases during follow-up. While the lack of follow-up EP studies limits the assessment of ablation durability in the presented study, evidence from other studies support the efficacy of a targeted ablation strategy.^10^ This strategy, which includes the elimination of SC-AIs and confirmation of bidirectional block along with ensuring non-inducibility immediately post-ablation, is likely more effective when compared with empiric ablation methods such as surgical cryoablation.^12^

There is a notable precedent within the field of paediatric and congenital EP for pre-interventional EP studies and a preventative ablation strategy. In Wolff–Parkinson–White (WPW) syndrome, the overall lifetime risk of sudden death is relatively low, ranging from 0 to 2.2 per 1000 patient-years. However, this risk is ‘front-loaded’ in the young, such that cardiac arrest may be the presenting symptom in otherwise asymptomatic children. Given the high likelihood of success and low risk of complications with catheter ablations, many paediatric electrophysiologists adopt an aggressive approach to asymptomatic WPW that includes invasive EP studies with pre-emptive ablation.^13^ Consider also the use of pre-operative EP assessment and pre-emptive catheter ablation of latent substrates in Ebstein malformation of the tricuspid valve before tricuspid valve replacement.^14^ This approach is deemed advisable due to concerns that tricuspid valve intervention may render arrhythmia substrates inaccessible and compromise the efficacy of catheter ablation. These examples reinforce the value of proactive strategies in identifying and eliminating arrhythmia conditions before they become problematic.

The amalgamation of these findings raises broader questions: Should routine catheter ablation be integrated into preventative management strategies for repaired TOF? Is prophylactic ablation of SC-AIs warranted for all repaired TOF patients? Will the successful ablation of latent VT substrates alter the lifetime risk for sudden death in this population? These considerations lie at the heart of ongoing debates in the field and form the basis for prospective multicentre studies, such as the CATAPULT-TOF study (Catheter Ablation of ventricular Tachycardia before transcatheter PULmonary valve replacement in repaired Tetralogy Of Fallot),^15^ which aim to explore the outcomes of a uniform pre-emptive strategy to eliminate VT substrates before transcatheter PVR in patients with TOF. The insights gleaned from such studies are poised to transform our approach to sudden death risk management for upcoming generations of repaired TOF individuals. The findings are expected to not only address the crucial interplay between technological advancements but also underscore the imperative for highly personalized approaches in the comprehensive care of patients with repaired TOF.

## Data Availability

No new data were generated or analysed in the writing of this editorial.
